# Bulimic behaviors in the tunisian general population: prevalence and associated factors

**DOI:** 10.1192/j.eurpsy.2024.1167

**Published:** 2024-08-27

**Authors:** A. Hadj Ali, M. Turki, M. A. Megdiche, S. Ellouze, G. Chakchouk, N. Halouani, J. Aloulou

**Affiliations:** Psychaitry B department, Hedi Chaker university hospital, Sfax, Tunisia

## Abstract

**Introduction:**

Bulimic behaviors (BB) are a major public health problem, due to their prognosis and serious psychological, somatic, and social consequences. The exact etiopathogenesis of BB is still poorly understood, and the literature suggests the interaction of multiple factors.

**Objectives:**

The aim of our study was to estimate the prevalence of BB in the Tunisian general population and to identify the associated risk factors.

**Methods:**

We conducted a cross-sectional, descriptive, and analytical study of Facebook group members, using an online questionnaire, from February 17, 2023, to May 26, 2023. All respondents over the age of 18 were included in the study. All participants filled out a socio-demographic questionnaire. Body mass index (BMI) was calculated from weight and height. The Bulimic Investigatory Test, Edinburgh (BITE) was used to screen and assess the intensity of bulimic behaviors.

**Results:**

A total of 528 responses were included in the study. The mean age of the sample was 33.3±11.95 years, and the M/F sex ratio was 0.41. Subjects were unmarried in 63.4% of cases, of low socio-economic status in 19.5%, with a university education in 75.2%, and with a psychiatric history in 25.6% of cases. The mean BMI was 25.15±4.98. The mean BITE score was 10.76±6.85, and 6.6% of our population were at high risk of developing BB.

In the bivariate study, female gender (p<0.001), unmarried marital status (p=0.001), university education (p<0.001), and the presence of a psychiatric history (p<0.001) were significantly associated with a high risk of developing BB. Moreover, the BITE score was negatively correlated with age (r=-0.231; p<0.001) and positively correlated with BMI (r=0.307; p<0.001).

**Conclusions:**

This study highlighted the magnitude of the risk of bulimic behaviors in the Tunisian general population and the need to set up programs to prevent and control these disorders.

**Disclosure of Interest:**

None Declared

